# Modulation of dielectric and antibacterial properties of Zn_0.5_Mn_0.5_O nanoparticles by post growth annealing method

**DOI:** 10.1016/j.heliyon.2024.e36035

**Published:** 2024-08-09

**Authors:** Cheng Zeng, Norah Salem Alsaiari, Muhammad Jawwad Saif, M. Junaid Dilshad, Tahir Mahmood Akhtar, Muhammad Isram, Adnan Ali, S. Younus, Norah Alomayrah, M.S. Al-Buriahi, K. Mahmood, M. Yasir Ali

**Affiliations:** a International Institutes of Medicine, The Fourth Affiliated Hospital of Zhejiang University School of Medicine, Yiwu, Zhejiang, 322000, China; b Department of Chemistry, College of Science, Princess Nourah bint Abdulrahman University, P .O. Box 84428, Riyadh, 11671, Saudi Arabia; c Department of Applied Chemistry, Government College University Faisalabad, Pakistan; d Department of Physics, Government College University Faisalabad, Pakistan; e University of Modena and Reggio Emilia, Italy; f Department of Physics, College of Sciences, Princess Nourah bint Abdulrahman University, P.O. Box 84428, Riyadh, 11671, Saudi Arabia; g Department of Physics, Sakarya University, Sakarya, Turkey

**Keywords:** ZnMnO nanoparticles, Hydrothermal method, LCR parameters, Antibacterial sensitivity

## Abstract

In this manuscript, we have investigated the dielectric and antibacterial potential of hydrothermally synthesized ZnMnO nanoparticles. The synthesized nanoparticles were annealed at various temperatures ranging from 450 to 650 °C with a step of 50 °C to modulate the structural, vibrational, dielectric, and antibacterial properties. XRD data confirmed the hexagonal structure of the synthesized samples and crystalline size was decreased to 4.8 nm at annealing temperature 600 °C. The lattice structure was further verified by Raman spectroscopy measurements, which strongly verified the XRD data due the presence of ZnMnO vibrational modes. The dielectric measurements revealed that the dielectric constant and los tangent were found to be increased with the increase annealing temperature and decreased with frequency, while a.c conductivity has an increasing trend with both parameters (temperature and frequency). The plot of real and complex parts of impedance against frequency demonstrated that both parameters decrease with the increased in frequency. But when we analyzed the behavior of the real part of impedance against the annealing temperature, a degradation in real part behavior is observed. The antibacterial activity of ZnMnO nanoparticles was determined by using the disc diffusion method against *E. coli* bacteria, which was grown on a Petri dish at room temperature for 24 h. This observation revealed that the samples annealed at 450 °C and 550 °C show remarkable antibacterial sensitivity as compared to other samples. It is concluded that crystalline size of 20 nm is found to be optimal value for good anti-baterial behavior.

## 1 Introduction

The use of nanotechnology and nanoparticles for dielectric application attracting academic's attention because of their potential applications in microelectronics and power density memory devices. The use of nano-materials for the treatment of bacterial infections is also gaining the interest of research community [[1], [2], [3], [4], [5], [6]]. For example, bacterial infections can be controlled by antibacterial vaccines, microbial diagnostics can be generated by bacterial detection systems, use of nanoparticles in antibacterial coatings for medical materials and devices [[7], [8], [9], [10], [11], [12], [13], [14], [15], [16]]. Therefore, synthesis of new and smart nanomaterials using simple and cost effective techniques can be advantageous to fabricate commercial devices.

Zinc oxide (ZnO) attracts the attention of researchers due to its wide range of applications. It is an n-type semiconductor material that belongs to group II-VI of the periodic table. The structure of zinc oxide is a wurtzite crystalline structure in which each atom of zinc is surrounded by four atoms of oxygen in tetrahedral coordination. The direct band gap energy for this material is 3.37 eV [8,9]. Its unique and specific properties such as wide band gap, high thermal conductivity, and high electron mobility make it a promising candidate for agriculture, pharmaceuticals, and ceramic applications [10]. It is also a well-known material for its applications in flexible gas sensors, light controlling, field effect transistors, and different electronic devices [[11], [12], [13]].

The medical, catalytic, electrochemical, electrical, and optical properties of ZnO can be controlled by introducing appropriate dopants with it [14]. Abdel-Baset *et,al* reported the fabrication of pure and doped nanoparticles with metals like Li, Mg, Cd, Fe, Cr, and Mn). They also observed that the crystalline structure of doped and un-doped samples is the same but pure ZnO nanoparticles showed paramagnetic behavior while doped with the above-mentioned materials exhibited ferromagnetic behavior at different levels of doping [15]. A decrease in the optical band gap energy with the increase in crystallite size when Mg was doped with ZnO was also observed [16].

ZnO nanoparticles doped with Mn exhibit hexagonal crystalline structure with a wide range of compositions and can make stable phase [17]. In previous studies different techniques were used in order to synthesize nanoparticles of ZnO such as wet chemical precipitation, sputtering, sol-gel, and hydrothermal methods [18]. Annealing is a powerful tool to modulate the structural, optical, dielectric and anti-bacterial activity. The crystalline size, intrinsic defects density and doping concentration can be easily controlled by annealing the grown samples at different temperatures.

In this manuscript, we have modulated the dielectric and anti-bacterial properties of hydrothermally synthesized ZnMnO nanoparticles by controlling the crystalline size. The crystalline size was varied in the range of 4–26 nm by post growth annealing technique at different temperatures (450–650 °C). It was observed that crystalline size of 20 nm is found to be optimal size for enhanced anti-baterial activity for E.Coli bacteria.

## 2 Experimental details

Hydrothermal synthesis method is very simple and cost effective method for the synthesis of nano-materials. This technique has many advantages over other synthesis methods i.e control over particle size and morphology, high purity of materials and low temperature requirements. In order to synthesize Zn_0.5_Mn_0.5_O nanoparticles by hydrothermal method, zinc chloride and manganese chloride of molecular weights 136.28 g/mol and 197.90 g/mol were used as precursors alongwith sodium hydroxide (NaOH) of molecular weight 39.9 g/mol and deionized water were used as solvents. The complete process started by dissolving 50 ml of deionized water with 3.40 g of zinc chloride. Similarly 4.94 g of manganese chloride was also dissolved in the same quantity of deionized water, and then both of the solutions were added in a glass beaker and continuously stirred. NaOH was added later to the solution to adjust the pH to 11. This solution was put into an autoclave which was placed in an oven at 200 °C for 24 h. The autoclave reactor was made of Stain less steel 304 with Teflon anodisation cell capacity 250 ml. After that, a fine powder of ZnMnO nanoparticles was obtained and dired at 100 °C. The ZnMnO powder was subjected to annealing process at various temperatures ranging from 450 to 600 °C for 1 h using a muffle furnace.

Crystalline structures of the prepared nanoparticles were studied by X-ray diffraction (D8 Advance, Bruker Germany) characterization technique. The active vibrational and rotational modes in the structure of the prepared sample were studied by Confocal Raman Spectrometer MN-STEX-PRI-100. To study the dielectric properties of prepared nanoparticles LCR measurements were performed by using an LCR meter. Finally, the antibacterial sensitivity of prepared ZnMnO annealed at different temperatures was observed by using the disc diffusion method against *E. coli* bacteria. The gram positive bacteria was obtained from our collaborators at GC University Faisalabad.

## 3 Results and discussions

**Fig. 1:**
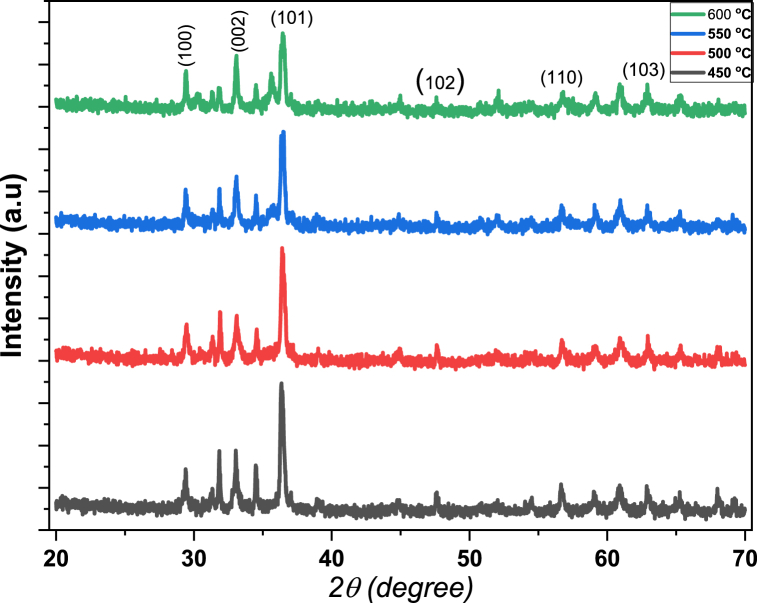
XRD pattern of ZnMnO nanoparticles.

XRD pattern of ZnMnO nanoparticles which were annealed at different temperatures is shown in Fig. 1. The data revealed that XRD data consisted of five major peaks of ZnMnO hexagonal structure at 2θ = 36.36°, 34.52° and 31.85°, 47.6°, 57.7° and 62.7° with miller indices (101), (002) (100), (120) and (110) respectively matching the results with (JCPDS card no. 36–1451) [19]. The crystalline size was found using XRD major peak calculated by the Scherrer formula given in equation (1)

(1) $$D=\frac{k\;\lambda }{\beta \;\cos\;\left(\theta \right)}$$

In the above equation, D is crystalline size, k = 0.94, λ is the wavelength of X-rays, β is the FWHM value and θ is the angle [20].

There are two other peaks in XRD graphs of all samples found at 33° and 29.4° these peaks may be due to the presence of manganese oxide nanoparticles in the prepared samples [21,22].

From XRD data it can be seen that when the annealing temperature increased from 450° to 500° the crystalline size of prepared nanoparticles increased from 20.5 nm to 26.5 nm which is due to the formation of larger grains in the nanoparticles by the movement of the oxygen atoms to the favorable positions and decrease in the crystalline defects [23]. With the further increase in annealing temperature the crystalline size decreases, The decrease in the crystalline size is associated with crystal discorder which is originated from thermal shocks [24]. After higher annealing, Mn atoms get high thermal energy and can easily penetrate to the interstitials sites. These interstitials sites act as intrinsic donor defects and may contribute two additional electrons to conduction band which resulted in the enhancement of carrier concentration. We have calculated some other parameters such as dislocation density, crystalline size and lattice parameters which are shown in Table 1.

**Table 1:** Effect of annealing temperature on crystalline size, microstrain and dislocation density calculated using major XRD peak.

Annealing Temperature (^o^C)	2theta (degree)	FWHM (Degree)	Crystalline size (nm)	Inter planer spacing (nm)	Microstrain (nm^−2^)	Dislocation density
450	36.36	0.41566	20.55	0.024	0.0017	0.0023
500	36.39	0.32224	26.51	0.024	0.0013	0.0014
550	36.52	0.42021	20.34	0.0241	0.0017	0.0024
600	36.43	1.77753	4.80	0.0240	0.0073	0.0432

**Fig. 2:**
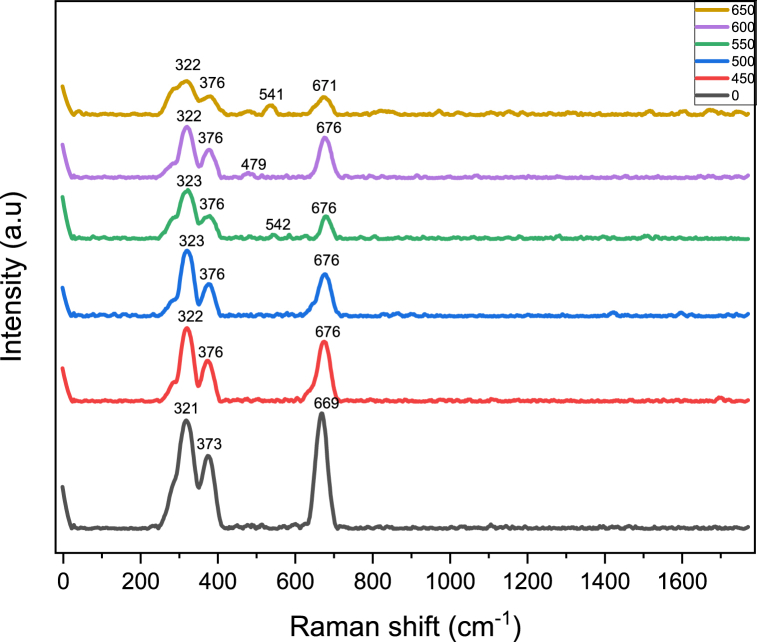
Raman spectroscopy results of ZnMnO nanoparticles annealed at different temperatures.

Raman spectroscopy is an easy research method to study the crystalline quality, orientations, chemical compositions, and lattice vibrations of the samples [25]. Raman Spectra of ZnMnO nanoparticles annealed at different temperatures are shown in Fig. 2. From the figure, it can be seen that all samples consist of Raman peaks at 320, 375, and 671 cm^−1^ [[23], [26]]. The origin of the occurrence of a peak around 676 cm^−1^ is due to the spinel phase of ZnMnO. The peaks at 669 cm^−1^ around 670 cm^−1^ occurred due to the incorporation of Mn^2+^ in the interstitials sites of ZnO [27,28]. The Raman peak around 540 cm^−1^ indicates the presence of crystalline defects and lattice disorder [28].

The complex dielectric permittivity can be expressed by following the relation

(2) $$\epsilon ˆ\left(*\right)=\epsilon^{\prime }\;-\;j\epsilon^{\prime \prime }$$

In equation (2) ϵ′ and ϵ" are the real and complex parts of dielectric permittivity respectively. The real part of permittivity expresses the energy stored and on the other hand imaginary part describes the energy dissipation under the application of an electric field. The dielectric constant ϵ_r_ can be calculated by using the following relation

(3) $$\epsilon_{r}=\frac{\epsilon^{\prime }}{\epsilon_{o}}$$

**Fig. 3:**
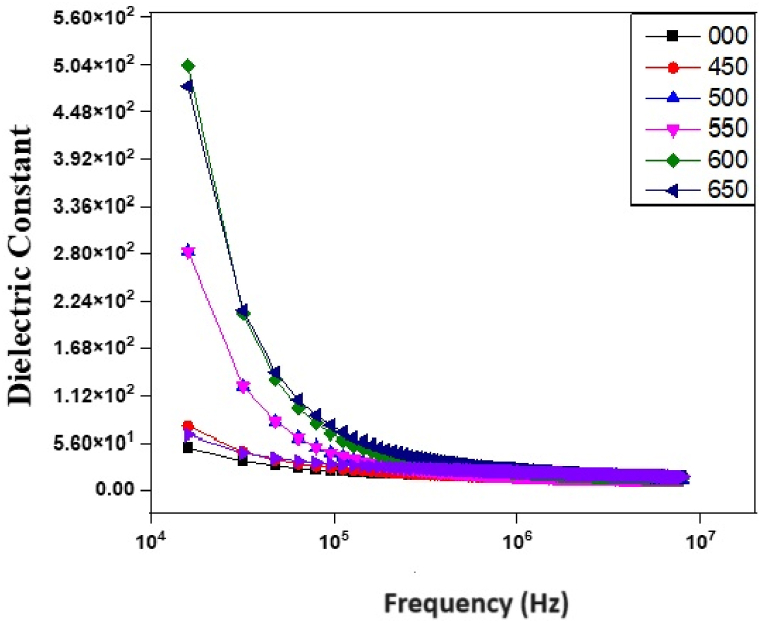
Dependence of dielectric constant on frequency and annealing temperature in ZnMnO nanoparticles.

In above equation $\epsilon_{o}$ is the primitivity of free space [29]. The variation of dielectric constant with frequency in ZnMnO nanoparticles annealed at 450 °C, 500 °C, 550 °C, 600 °C, and 650 °C is shown in Fig. 3. It can be observed that the dielectric constant decreases rapidly with the increase in frequency but at higher frequencies, it becomes almost constant. This behavior of the dielectric constant can be explained by Maxwell-Wager's interfacial model and Koop's phenomenological theory [30]. The decrease in the dielectric constant with the increase in frequency is attributed to the reduction of the space charge polarization effect. However, the presence of space charge polarization at grain boundaries at lower frequencies leads to higher values of the dielectric constant [31]. From Fig. 3 it can also be seen that with the increase in annealing temperature dielectric constant increases which is due to the increase of crystal defects. Such crystal defects may originated at higher annealing temperature because at higher annealing temperature, some of Mn atoms may get thermal energy and penetrate to the interstitials sites. Mn atoms interstitials sites acted as intrinsic donor defects. These donor defects leads to an increase in the thickness of grain boundaries so the reduction of space charge polarization occurs due to which dielectric constant decreases [24,32]. The contribution of space charge polarization is stronger in nanomaterials which is why the dielectric constant of nanomaterials is high at low frequencies. At higher frequencies the space charge polarization cannot keep up with electric field resulting a decrease of dielectric constant and loss tangent with the increasing frequency and annealing temperature [33].

Loss tangent or Dielectric loss is an important factor that describes the dissipation of energy into heat during the orientation of dipoles in an electric field against the resistance present due to inertia in dielectric systems. The mathematical expression of this factor is given as [34].

(4) $$\tan\;\delta =\frac{\epsilon^{\prime \prime }}{\epsilon^{\prime }}$$

**Fig. 4:**
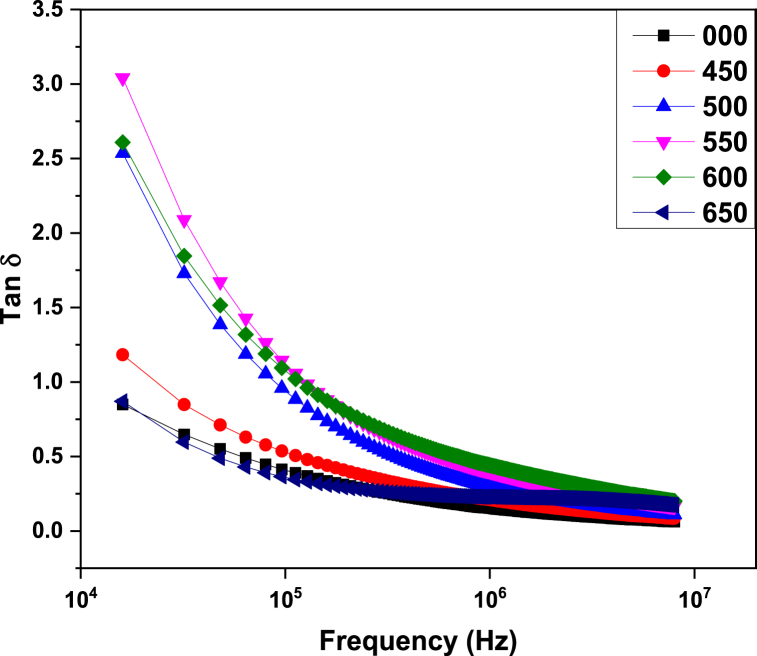
Variation of loss tangent with frequency in ZnMnO nanoparticles annealed at different temperatures using a muffle furnace.

Fig. 4 represents the behavior of loss tangent with frequency for synthesized samples. A decrease in the loss tangent with the increase in frequency can be observed from this figure. This variation in loss tangent with frequency can be explained by Koop's model and Maxwell Wager's model. According to these models, the loss tangent is greater at lower frequencies because of the larger time duration between polarization and depolarization while at higher frequencies dipole moment becomes independent of the electric field [24].

**Fig. 5:**
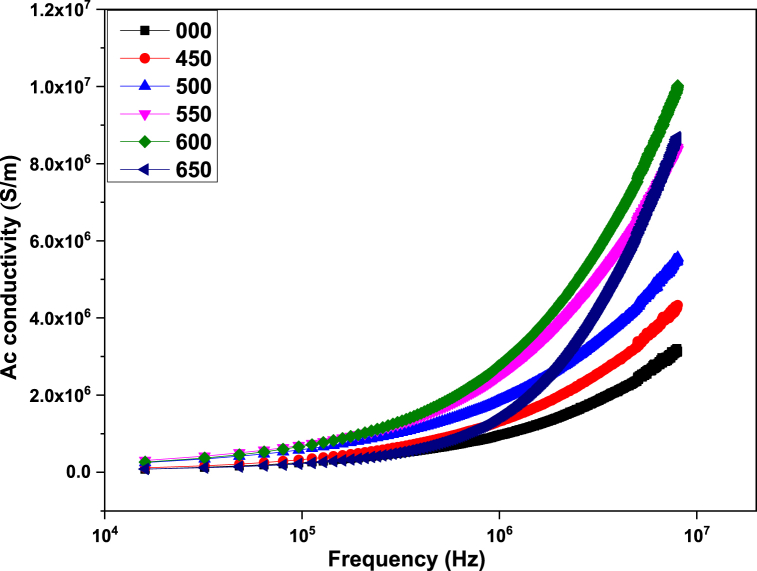
Dependence of ac conductivity on frequency in ZnMnO nanoparticles.

Fig. 5 indicates the variation in ac conductivity with frequency and annealing temperature of ZnMnO nanoparticles annealed at various temperatures. The ac conductivity of the material is given by the flowing mathematical expression.

(5) $$\sigma_{ac}=\epsilon^{\prime }\epsilon_{o}\omega \;\tan\;\delta$$

In equation (5) ω is the angular frequency of ac signal which can be described as ω = 2πf [29]. From the figure it can be seen that ac conductivity increases with the increase in frequency. The fact behind the low ac conductivity at lower frequencies is that except for a small number, charger carriers cannot tunnel through a potential barrier between grains due to grain boundaries. At higher frequencies charge carriers gain sufficient energy to overcome this potential barrier so ac conductivity increases [35,36]. The ac conductivity depends on various factors such as secondary phases, interstitials, vacancies, and charge carrier concentration [37]. From figure (5) it can also be observed that with the increase in annealing temperature, conductivity increases. This behavior is due to presence of Mn based interstitials donor defects. At higher annealing temperature, some of the Mn atoms may occupy the interstitials sites by getting high thermal energy. These interstitials donor defects resulted in the carrier concentration and consequently the a.c conductivity. Which is due to the increase in Mn contents [29,36].

Impedance is an important factor that describes the electrical properties of materials. The complex relation of impedance can be described by the following equation.

(6) Z* = *Z' -jZ"*

**Fig. 6:**
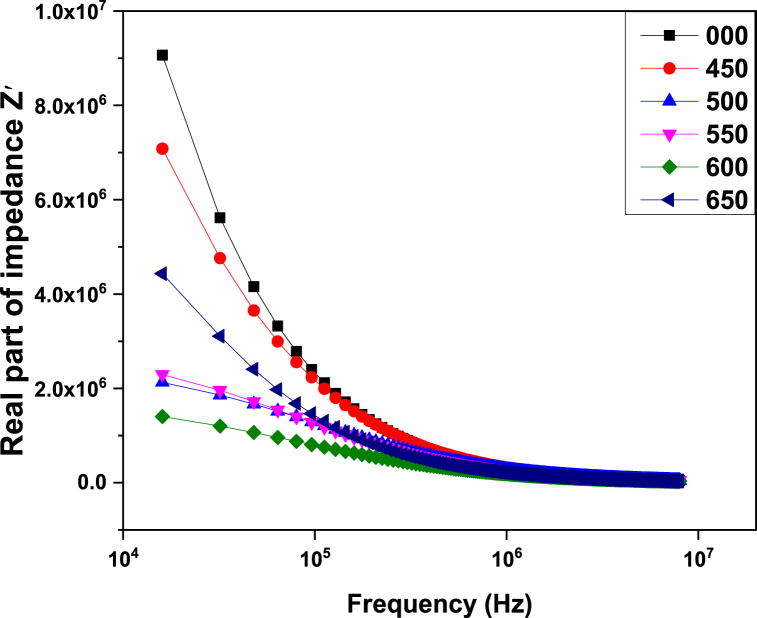
Variation of the real part of impedance with frequency for ZnMnO nanoparticle.

**Fig. 7:**
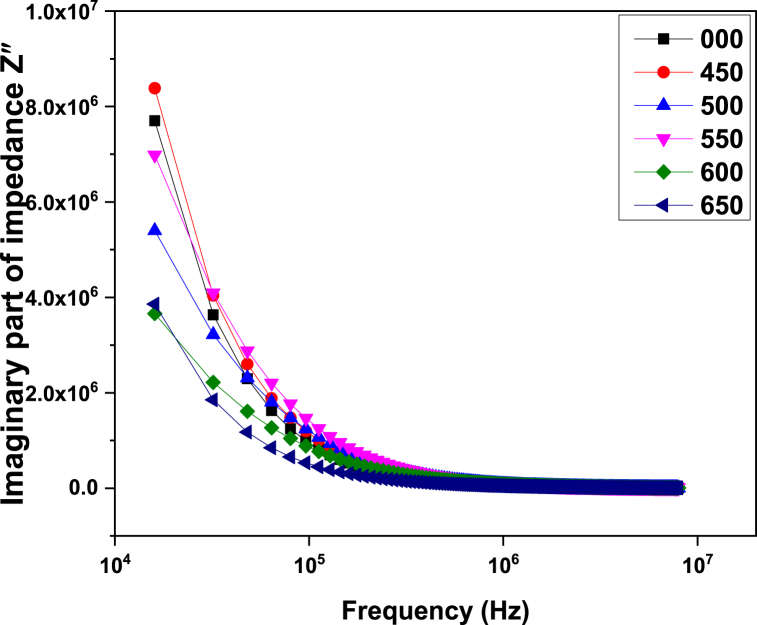
Variation of the imaginary part of impedance with frequency for ZnMnO nanoparticles.

In equation (6) *Z′* and *Z”* are the real and imaginary parts of impedance [38]. Figure (6) describes the dependence of the real part of impedance on frequency. From the figure, it can be seen that with the increase in frequency, real part of impendenc decreases which corresponds to the fact that at lower frequencies ac conductivity is smaller, and with the increase in frequency ac conductivity increases so the charge carriers have to face lower resistance [38]. From figure (7) it can be observed that the imaginary part of complex impedance shows almost the same behavior as the real part.

**Fig. 8:**
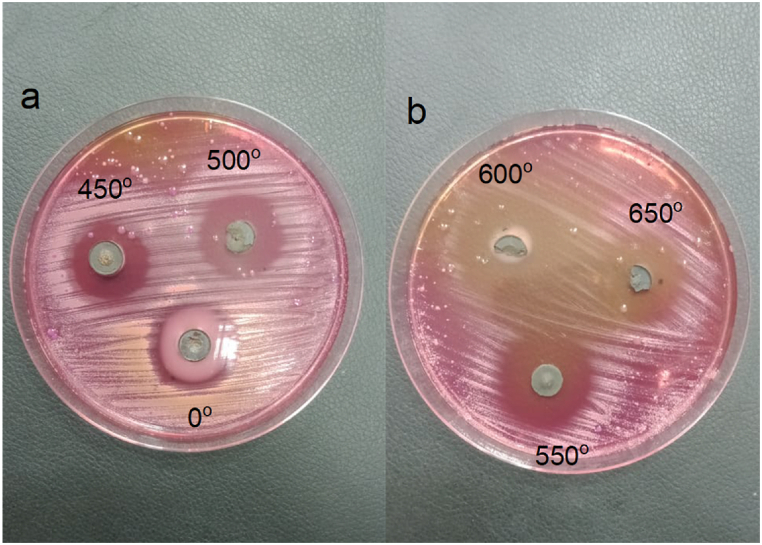
Antibacterial activity of ZnMnO nanoparticles annealed at (a) 0 °C, 450 °C, and 500 °C (b) 550 °C, 600 °C and 650 °C.

The antibacterial activity of ZnMnO nanoparticles annealed at various temperatures against *E. coli* was determined using the disc diffusion method is shown in figure (8). The above-mentioned bacteria were grown on a Petri dish at room temperature for 23 h. From the figure, it can be seen that the ZnMnO nanoparticles annealed at 450 °C and 550 °C show remarkable antibacterial sensitivity against *E. coli*. The samples that were annealed at 600 °C and 650 °C show a little bit smaller diffusion zones which indicates that the antibacterial sensitivity decreases with the increase in annealing temperature. We have linked the anti-bacterial properties with the crystalline size of material. The antibacterial activity of ZnMnO nanoparticles against E.coli decrease with the increase in annealing temperature. This type of behavior of antibacterial activity was reported in literature [39]. The optimal value of crystalline size was found to be 20 nm for enhanced anti-bacterial properties. The antibacterial activity of nanomaterials depends upon many other factors such as crystalline size, surface area, aspect ratios, ROS generations etc. The nanomaterials with smaller crystalline size exhibit good antibacterial activity than those with larger crystalline size [40]. During this research the sample which was annealed at 450° C exhibited best antibacterial activity because its crystalline size is 20.5 nm.

**Fig. 9:**
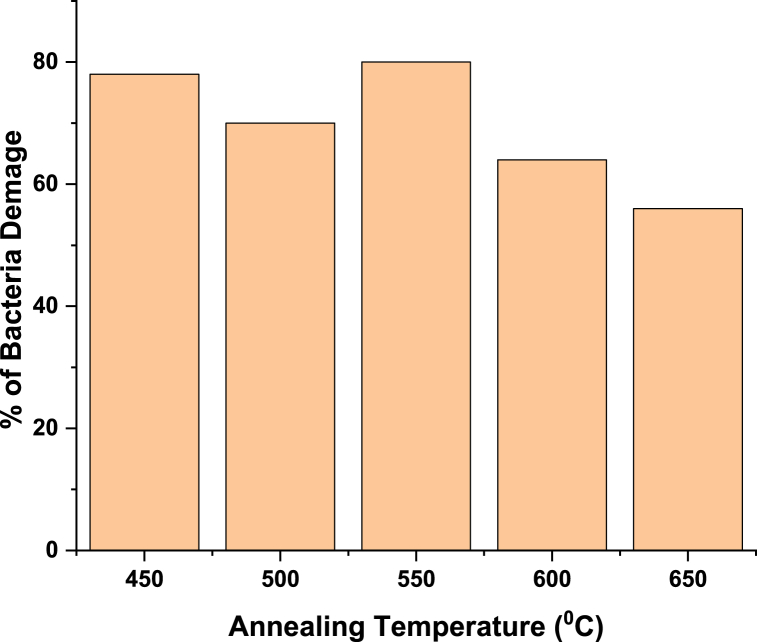
Effect of annealing temperature on the % damage of E.Colie

The histogram shown in Fig. 9 is showing the percentage of E.Coli bacteria damage with annealing temperature. The graph is again verifying that a sample which has specific crystalline size of 20 nm has shown the maximum percentage of E.Colia damage.

## 4 Conclusions

This article reports the synthesis of ZnMnO nanoparticles by hydrothermal method and post growth annealed at different temperatures ranging 450–650 °C. All the samples exhibit hexagonal crystalline structure and the crystalline size for 450 and 500 °C was found to be 20 nm. Dielectric constant of all synthesized samples decreased with the increase in frequency. From the dielectric measurements, it was observed that with the increase in annealing temperature causes dielectric constant and loss tangent decreased. The ac conductivity measurements of synthesized samples show that with an increase in frequency and annealing temperature, ac conductivity increases. From the impedance analysis, it was observed that the real and complex parts of the impedance of synthesized samples decrease with the increase in frequency due to the increase in ac conductivity. The antibacterial activity of the sample was performed by the disc diffusion method against *E. coli*. The observations indicate that the sample which was annealed at 450 °C and 550 °C showed comparatively better antibacterial activity. This behavior of antibacterial activity is related the optimal value of crystalline size which is found to be 20 nm when compared with the previous reports in the literature.

## Data availability statement

The machine/raw data will be provided upon request.

## CRediT authorship contribution statement

**Cheng Zeng:** Analysis. **Norah Salem Alsaiari:** manuscript writting. **Muhammad Jawwad Saif:** Conceptualization. **M. Junaid Dilshad:** Data curation. **Tahir Mahmood Akhtar:** Formal analysis. **Muhammad Isram:** funding. **Adnan Ali:** Investigation. **S. Younus:** Project administration. **Norah Alomayrah:** proof reading. **M. S. Al-Buriahi:** discussion. **K. Mahmood:** Conceptualization. **M. Yasir Ali:** Methodology.

## Declaration of competing interest

The authors declare that they have no known competing financial interests or personal relationships that could have appeared to influence the work reported in this paper.
